# Hematocolpos as a Result of Delayed Treatment of Acute Straddle Injury in an Adolescent Girl

**DOI:** 10.1155/2016/1987690

**Published:** 2016-02-22

**Authors:** Hae Jin Hwang, Hyun Wook Lim, Young Shin Han, Jeong In Choi, Min Jeong Kim

**Affiliations:** ^1^Department of Obstetrics and Gynecology, Bucheon St. Mary's Hospital, College of Medicine, The Catholic University of Korea, 327 Sosa-ro, Wonmi-gu, Bucheon-si, Gyeonggi-do 14647, Republic of Korea; ^2^Department of Radiology, Bucheon St. Mary's Hospital, College of Medicine, The Catholic University of Korea, 327 Sosa-ro, Wonmi-gu, Bucheon-si, Gyeonggi-do 14647, Republic of Korea

## Abstract

Accidental genital trauma is most commonly caused by straddle-type injuries and is usually treatable by nonoperative management, and most of the injuries have a good prognosis. When the bleeding occurred due to straddle injury in adolescent girl, experienced gynecological examination and treatment were very important. We experienced a case of straddle injury to the posterior fourchette that caused acute hematocolpos due to delayed adequate treatment with hypotension and acute abdomen in an adolescent girl. This case shows the importance of careful and accurate physical and gynecological examination and adequate and prompt treatment of genital trauma in adolescent girls.

## 1. Introduction

Nonsexual, nonobstetric gynecologic injuries in young girls can be broadly categorized into 3 types based on the mechanism of injury: (1) straddle injury (the most frequent mechanism), (2) nonstraddle blunt injury, and (3) penetrating trauma [1]. Genital injuries in young girls raise concerns regarding the possibility of sexual abuse, and the literature characterizing these injuries has focused on abuse-related causes [1].

We experienced a case of genital trauma after straddle injury that caused hematocolpos with acute abdominal pain and hypotension in a 14-year-old girl, and it emphasized the importance of careful examination and prompt treatment of genital trauma in adolescent girls.

## 2. Case Presentation

A 14-year-old girl visited our Emergency Department with vaginal bleeding. She fell down at home and suffered a straddle injury to the perineal area. She had no underlying disease, and menarche had occurred two months ago.

Her parents stated that there was about 400 cc of blood loss from the laceration and they were not cooperative with physical examination of her genital area because of pain and resistance to gynecological examination in emergency department. Her vital signs on arrival were as follows: blood pressure, 139/64 mmHg; heart rate, 109/min; respiratory rate, 20/min; temperature, 36.5°C; and the hemoglobin/hematocrit was 12.1/35.4 mg/dL. The patient and her parents did not give their consent for an invasive procedure; hence emergency team decided to perform expectation therapy under compression procedure.

**Figure 1:**
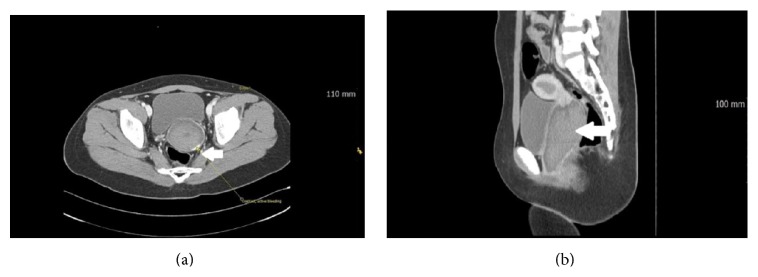
Computed tomographic findings. (a) Axial view. Arrow shows contrast extravasation and active bleeding in vagina. (b) Blood-filled vagina means hematocolpos in sagittal view.

After two hours, her abdominal pain was aggravated throughout the whole abdomen. Blood pressure was 90/60 mmHg, pulse rate was 120/min, respiratory rate was 20/min, and body temperature was 37.2°C. Emergency team decided to perform pelvic computed tomography (CT) scans for identifying internal organ damage after injury 2 hours later. The pelvic CT showed hematocolpos with active vaginal bleeding. Her vagina was filled with high attenuation fluid, implying blood, and extravasation of the contrast material from 4-5 o'clock position of the vagina was observed. It suggested that active bleeding was ongoing (Figures 1(a) and 1(b)).

**Figure 2:**
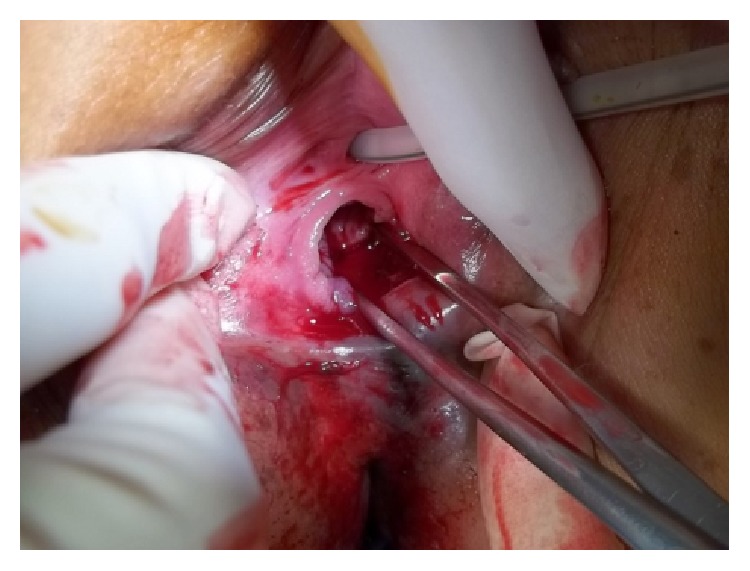
Laceration of the hymen and posterior fourchette.

They reported gynecological team and we thoroughly inspected the perineal area and found an approximately 2 cm laceration of the posterior fourchette and a laceration at 5 o'clock position of the hymen. Bleeding was ongoing. We decided to perform a surgical procedure. More than 300 mL of blood and clots were aspirated through the vagina and an approximately 2 cm trigonal shaped laceration was noted on the posterior fourchette (Figure 2) and primary closure was performed with Vicryl 3-0.

Two days after the surgery, she was discharged without any complications. We examined the vagina and perineal area in the outpatient clinic. All of the scars were clean, and she showed no sequelae.

## 3. Discussion

In girls aged <15 years, genital trauma is the most common gynecologic cause of emergency department visits [2]. Accidental genital trauma is most commonly caused by straddle injuries [1]. The types of injuries seen in the genital area vary from abrasion to contusion, lacerations, and hematoma. Injuries to the perineum occur in more than 20% of the patients, and injuries to the posterior fourchette and hymenal disruption (which are typically associated with sexual assault) are uncommon. In most of the cases, these injuries are amenable to expectant management but sedation or operative intervention may be needed when complete examination cannot be performed or when the injuries are extensive [1].

The history given by the child and the caregivers and/or any witness is the most important element in the assessment of a child presenting with a genital injury [3].

Our case was of hematocolpos caused by a perineal straddle injury in an adolescent girl due to delayed diagnosis and treatment. At first, the patient had an uncooperative attitude towards gynecological examination; hence, a compression dressing was applied and only close observation was performed in the emergency department. However, bleeding was ongoing, and there was newly developed abdominal pain because of obstruction of bleeding caused compression of injured site. Pulse rate was increased, and hypotension developed after 2 hours. Pelvic CT scans showed a blood-filled vagina, and active bleeding was ongoing. We decided to perform an emergency operation for achieving hemostasis and extraction of blood from the vagina.

Occasionally, girls are reluctant to undergo gynecological examination and it may be more difficult to perform gynecological examination in the emergency department, since adolescents have a higher level of anxiety about their injuries than infants. When patient distress precludes an examination, or when extensive injuries are obvious or suspected, evaluation under conscious sedation or general anesthesia is necessary to ensure complete examination [1].

Sexual abuse should always be considered and ruled out through a detailed history that correlates with the physical examination findings, especially in case of injuries to the posterior fourchette or the hymen, which are uncommon in genital trauma among adolescent girls [1].

Many studies demonstrate that most accidental genital injuries can be managed conservatively because an adequate assessment and examination in the emergency department without surgical management are possible in almost 80% of cases of accidental female genital trauma [3].

Conservative management should enable the child to void spontaneously. Healing can be achieved by using the recommended conservative measures, including sitz bath and reduction in the physical activity during the critical healing phase, especially in the first 48 to 72 hours [3–5]. Reduction in the activity level helps to ensure that the area is not reinjured before considerable healing has taken place [5]. Gynecologists should be familiar with the evaluation and management of adolescent girls with a history of genital trauma, and they should be trained to manage this condition.

Although most of the injuries can be treated conservatively, we would like to emphasize that physicians should keep in mind that genital trauma in children and adolescents can cause an emergency as observed in our case.
